# Niclosamide is a potential candidate for the treatment of chemo-resistant osteosarcoma

**DOI:** 10.1590/1678-4685-GMB-2022-0136

**Published:** 2023-02-03

**Authors:** Xiaoling Huang, Wei Wang, Yong Li

**Affiliations:** 1 Wuhan Fourth Hospital, Department of Pulmonary and Critical Care Medicine, Wuhan, Hubei, China.; 2 Wuhan Fourth Hospital, Department of Orthopaedics, Wuhan, Hubei, China.; 3 Wuhan Fourth Hospital, Department of Pharmacy, Wuhan, Hubei, China.

**Keywords:** Osteosarcoma, niclosamide, β-catenin, chemo-resistance

## Abstract

Chemotherapy is the main treatment option for advanced osteosarcoma, which is the most common type of primary bone malignancy. However, patients develop resistance rapidly and many succumb to the disease. Niclosamide, an anthelmintic drug, has been recently identified to display potent and selective anti-cancer activity. In this work, we show that niclosamide at sub-micromolar concentrations inhibits proliferation and migration, and induces apoptosis in both parental and chemo-resistant osteosarcoma cells, with much less toxicity in normal osteoblastic cells. Interestingly, chemo-resistant osteosarcoma cells are more sensitive to niclosamide compared to parental cells. We further identify that inhibition of β-catenin is the underlying mechanism of niclosamide’s action in osteosarcoma cells. In addition, we reveal that chemo-resistant osteosarcoma cells display increased β-catenin activity compared to parental cells, which might explain the hypersensitivity of chemo-resistant cells to niclosamide. Our work provides pre-clinical evidence that niclosamide can be repurposed for treating osteosarcoma. Our findings also suggest the therapeutic value of β-catenin to overcome osteosarcoma chemo-resistance.

## Introduction

Osteosarcoma is the most common malignant bone tumor in children and adolescent with poor prognosis. The treatment for osteosarcoma is limited, including neoadjuvant chemotherapy (pre-operative), surgical resection and adjuvant chemotherapy (post-operative) (Harrison et al., 2018). The most commonly used chemotherapy regimen is the combination of high-dose doxorubicin, cisplatin, and methotrexate, which achieved 5-year survival rates of approximately 60% (Zhang et al., 2018). Chemo-resistance is a significant problem for majority of patients and causes 90% of treatment failure (Marchandet et al., 2021). The underlying mechanisms are not well-understood, but postulated to be associated with regulation of drug transporter proteins, inhibition of tumor suppressors, genomic instability and activation of oncogenic signaling (Lilienthal and Herold, 2020). Identifying alternative therapeutic strategies that target molecular mechanisms of chemo-resistance are essential to improve clinical outcome of osteosarcoma. 

Niclosamide is a widely-used oral anthelminthic drug to treat human parasitic infections. Although the mechanism of niclosamide’s action has not been well elucidated, studies reveal that it is linked to uncoupling of oxidative phosphorylation (Weinbach and Garbus, 1969; Tanowitz et al., 1993). Substantial evidence has shown that niclosamide is a multifunctional drug that can be repurposed for other diseases, particularly cancer (Chen et al., 2018). We and others demonstrate that niclosamide induces apoptosis and cell cycle arrest for numerous cancers that include ovary, intestinal, liver, cervix, kidney, breast and blood (Khanim et al., 2011; Lu et al., 2011; Osada et al., 2011; Arend et al., 2014; Zhao et al., 2016; Chen *et al.*, 2017; Wang C et al., 2018). The mechanism of action for niclosamide across different cancers is not universal but seems to be cancer type-specific. Wnt/β-catenin, ATF3, PERK, eIF4E, NK-ĸB, STAT3, and mTOR have been reported to be involved in niclosamide’s action in cancer cells (Arend *et al.*, 2016; Liu Z et al., 2016; Weng et al., 2016; Wang *et al.*, 2019; Wei *et al.*, 2021). Several studies also highlight the inhibitory effects of niclosamide on osteosarcoma cells and xenograft mouse model (Li *et al.*, 2015; Reddy et al., 2020; Yi et al., 2021; Yeh et al., 2022). However, it is not known whether niclosamide is effective against chemo-resistant osteosarcoma. 

In this work, we first established the cisplatin-resistant and doxorubicin-resistant osteosarcoma cell lines by pro-longed exposure of parental cells to chemotherapy drugs. We next systematically investigated the efficacy of niclosamide on parental and chemo-resistant cells. Finally, we determined the underlying mechanism of niclosamide’s action in chemo-resistant cells and identified the therapeutic target to overcome osteosarcoma chemoresistance. 

## Material and Methods

### Compounds, cell culture and MTT assay

Niclosamide (Catalog No. S3030, HPLC > 99%), lithium chloride (LiCl, Catalog No. E0153, HPLC > 99%), doxorubicin (Catalog No. S1208, HPLC > 99%) and cisplatin (Catalog No. S1166, HPLC > 99%) were all purchased from Selleckchem and reconstituted as per manufacturer’s instructions. Human osteosarcoma cell lines MG-63, Saos-2 and HOS-143B, and human osteoblastic cell line OB-6 were obtained from the Cell Bank of Shanghai Institute of Biological Science. Primary human osteoblasts and hepatocytes were obtained from Lonza and maintained in medium as per manufacturer’ recommendations. Osteosarcoma cell lines were authenticated using short tandem repeat profiling analysis (Precision Genome Biotechnology Inc.) and all cells were examined for mycoplasma using MycoAlert™ mycoplasma detection kit (Lonza). Cells were cultured in ATCC-formulated McCoy’s 5a Medium Modified (Catalog No. 30-2007). Cell growth/viability studies were carried out using CellTiter® 96 Aqueous One Solution cell proliferation assay (Promega). 

### Generation of resistant cell lines

MG-63-dox-r and HOS-143B-cis-r cells were established by prolonged exposure of parental cells to doxorubicin and cisplatin, respectively. MG-63 and HOS-143B cells were initially cultured in medium containing 10 nM doxorubicin and 0.1 µM cisplatin. The concentrations were gradually increased by 1.5- to 2-fold each time and resistant cells were finally maintained in medium containing 20 µM doxorubicin or 100 µM cisplatin. 

### Western blotting 

Drug treated cells were washed with ice cold PBS on ice and lysed in RIPA lysis buffer (Life Technologies) supplemented with protease inhibitor cocktail (Roche). Protein concentration were determined using BCA protein assay kit (Pierce). 20-40 µg of protein lysate per sample was resolved on SDS-polyacrylamide gels and transferred onto PVDF membrane (Millipore). Western blotting was performed using antibodies recognizing β-catenin and β-actin (Santa Cruz Biotechnology). Signals were detected on Li-COR Odyssey imaging system. 

### Measurement of proliferation and apoptosis

After 3 days drug treatment, cell proliferation and apoptosis were determined using BrdU proliferation assay kit (Abcam) and Annexin V-FITC and 7-AAD (Beckman Coulter) as described in our previous study (Li et al., 2018).

### Measurement of migration 

Migration assay was performed using the Boyden chamber. Cells together with drugs were pre-incubated in RPMI1640 medium supplemented with 2% FBS for 30 min at 37 ºC before seeding onto the cell culture inserts. Cells migrated through 8-µm pore size polycarbonate membrane filters in Falcon cell culture insert toward to 10% FBS placed in the lower chamber. After 6 hours, non-migrating cells were removed with a cotton swab. Migrated cells on the lower surface of the culture inserts were stained with 0.4% crystal violet (Sigma). 

### Real time (RT)-PCR 

RNA extraction, cDNA preparation and quantitative RT-PCR were carried out using the same protocol as reported in our past study (Li et al., 2018). The primers are 5’-AAT GAA AAG GCC CCC AAG GT AGT TAT-3’ and 5’-GTC GTT TCC GCA ACA AGT CCT CTT-3’ for MYC; and 5’-CCG TCC ATG CGG AAG ATC-3’ and 5’-ATG GCC AGC GGG AAG AC-3’ for CYCLIN D1. All RT-PCR data presented were normalized using GAPDH. 

### Luciferase reporter assay

1x10^4^ cells were seeded in a 24-well plate and transfected with a M50 Super 8x TOPFlash plasmid (a kind gift from Dr. Randall Moon) (Veeman et al., 2003) together with pRenillaTK in a 1:10 ratio and the respective plasmid mentioned in the experiment as a total of 250 ng DNA. After 24 hours, cells were treated with drugs. After 24 hours drug treatment, dual luciferase reporter assays (Promega) were done as per the manufacturer’s instructions. TOP was calculated after normalizing luciferase values to renilla values. 

### Statistical analyses

All data are expressed as mean ± SD. One-way analysis of variance followed by Tukey’s HSD test was conducted for multiple comparisons. Statistical analyses were performed by unpaired Student’s t test, with p-value < 0.05 considered statistically significant.

## Results

### Niclosamide is selectively active against parental and chemo-resistant osteosarcoma cells

**Figure 1 -:**
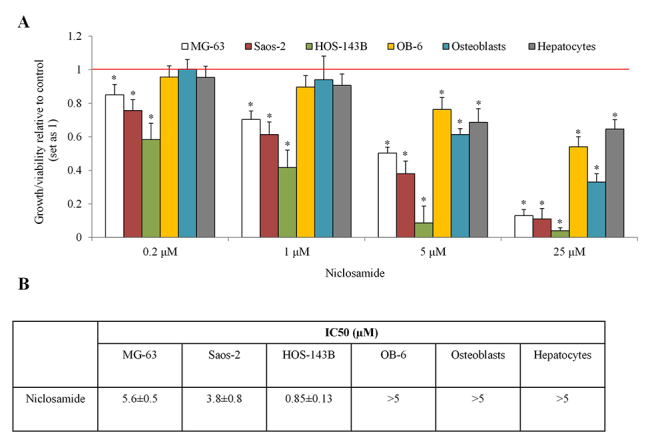
Niclosamide is preferentially active against osteosarcoma cells. (A) Niclosamide at 0.2, 1, 5 and 25 µM dose-dependently decrease growth/viability of multiple osteosarcoma cell lines: MG-63, Saos-2 and HOS-143B. Niclosamide at 5 and 25 µM but not 0.2 and 1 µM significantly decrease growth/viability of OB-6, osteoblastic and hepatocyte cells. After 3 days treatment, cell growth/viability was measured using CellTiter® 96 Aqueous One Solution cell proliferation assay. *, p<0.5, compared to control. Control was set up as 1. Unpaired Student’s t test was conducted. (B) IC_50_ of niclosamide on different types of cells. IC_50_ was determined using GraphPad Prism software.

To examine whether niclosamide has selective anti-osteosarcoma activity, we treated a panel of human osteosarcoma cell lines and human OB-6 osteoblastic cells, followed by measuring the level of NAD(P)H-dependent dehydrogenase enzymes which indicate overall cell growth and viability. Niclosamide at 0.2 µM started to inhibit growth/viability and at 25 µM caused near-total growth arrest and viability inhibition in all tested osteosarcoma cell lines, including MG-63, Saos-2 and HOS-143B (Figure 1A). Consistent with our previous findings that niclosamide is less toxic to normal cells than tumor cells (Liu Z et al., 2016), niclosamide at the same concentration either did not affect or inhibited growth and viability of OB-6, human primary osteoblast, and hepatocyte in a significantly lesser degree than osteosarcoma cells (Figure 1A). The IC50 of niclosamide on normal cells including OB-6, osteoblast and hepatocyte are higher than osteosarcoma cells (Figure 1B).

**Figure 2 -:**
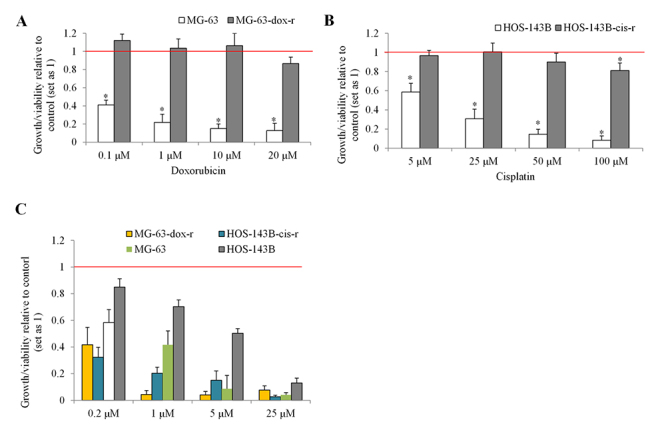
Niclosamide is preferentially active against chemo-resistant osteosarcoma cells**.** (A) The growth/viability of MG-63 and MG-63-dox-r cells in the presence of doxorubicin. (B) The growth/viability of HOS-143B and HOS-143B-cis-r cells in the presence of cisplatin. After 3 days treatment, cell growth/viability was measured using CellTiter® 96 Aqueous One Solution cell proliferation assay. (C) Niclosamide at 0.2, 1, 5 and 25 µM dose-dependently decreases growth/viability in MG-63, HOS-143B, MG-63-dox-r and HOS-143B-cis-r cells. *, p<0.5, compared to control. Control was set up as 1. Unpaired Student’s t test was conducted.

To challenge niclosamide’s suitability for the treatment of osteosarcoma, we generated chemo-resistant osteosarcoma cell lines and performed the same analysis. MG-63, Saos-2 and HOS-143B were cultured in the presence of doxorubicin or cisplatin at gradually increasing concentrations. Although generation of chemo-resistant Saos-2 was unsuccessful, we established doxorubicin-resistant MG-63 (MG-63-dox-r) and cisplatin-resistant HOS-143B (HOS-143B-cis-r) cells. The complete resistance of MG-63 and HOS-143B to doxorubicin and cisplatin was confirmed by detecting the level of growth/viability in both parental and chemo-resistant osteosarcoma cell lines after cisplatin or doxorubicin treatment (Figure 2A andB). In addition, niclosamide at sub-micromolar concentrations potently decreased MG-63-dox-r and HOS-143B-cis-r cells (Figure 2C). Taken together, our data clearly show that niclosamide potently and selectively inhibits osteosarcoma cells. 

### Niclosamide inhibits proliferation, induces apoptosis and suppresses migration in both parental and chemo-resistant osteosarcoma cells

**Figure 3 -:**
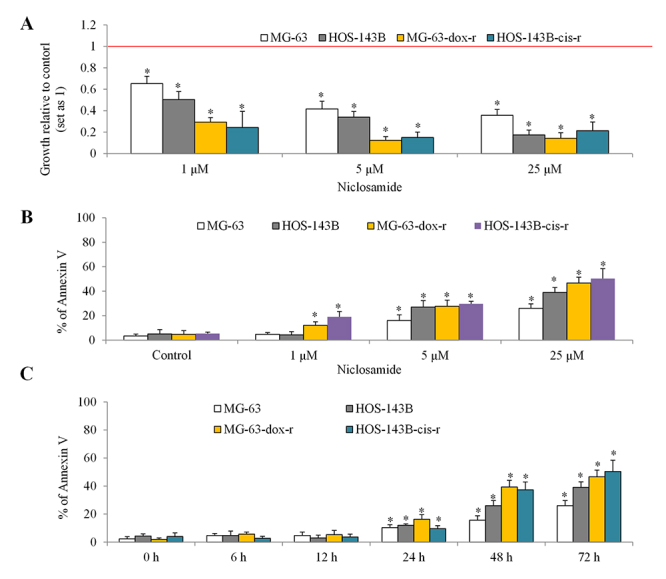
Niclosamide decreases proliferation and induces apoptosis in parental and chemo-resistant osteosarcoma cells. (A) Niclosamide at 1, 5 and 25 µM significantly decreases proliferation in MG-63, HOS-143B, MG-63-dox-r and HOS-143B-cis-r cells. (B) Niclosamide at 1, 5 and 25 µM significantly induces apoptosis in MG-63, HOS-143B, MG-63-dox-r and HOS-143B-cis-r cells. Cells were treated with drugs for 72 h prior to proliferation (as measured by BrdU incorporation) and apoptosis (as measured by flow cytometry of Annexin V) assays. (C) Time course analysis of apoptosis on niclosamide on parental and chemo-resistant osteosarcoma cells. *, p<0.5, compared to control. Unpaired Student’s t test was conducted.

**Figure 4 -:**
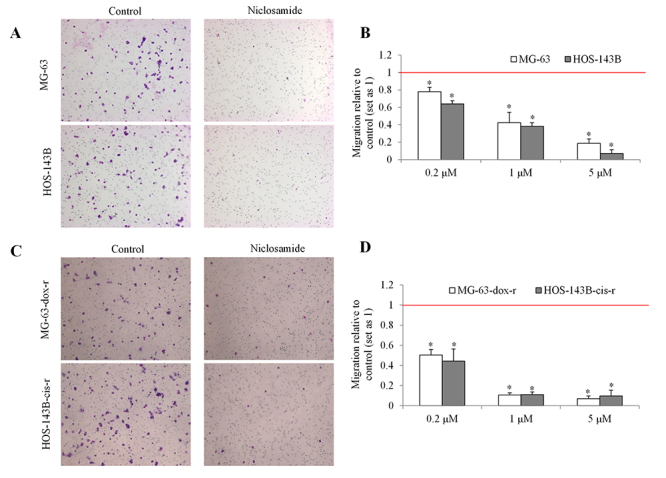
Niclosamide decreases migration in parental and chemo-resistant osteosarcoma cells. (A and C) Representative images of migration of MG-63, HOS-143B, MG-63-dox-r and HOS-143B-cis-r cells in the absence (control) and presence of niclosamide (5 µM). (B and D) Quantification of osteosarcoma cell migration exposed to different concentrations of niclosamide. *, p<0.5, compared to control. Control was set up as 1. Unpaired Student’s t test was conducted.

To elucidate what cellular activities of osteosarcoma that niclosamide inhibits, we performed proliferation, apoptosis and migration assays in both parental and chemo-resistant osteosarcoma cells after niclosamide treatment. As assessed by BrdU incorporation, we found that niclosamide at 1 to 25 µM inhibited proliferation of MG-63 and HOS-143B cells (Figure 3A). Interestingly, niclosamide also inhibited proliferation of MG-63-dox-r and HOS-143B-cis-r cells, and furthermore that niclosamide at the same concentrations (eg, 1 µM and 5 µM) inhibited 70~85% growth in chemo-resistant cells and 35~65% growth in parental cells (Figure 3A). As assessed by flow cytometry with Annexin V, niclosamide at 1 µM, 5 µM and 25 µM induced ~5%, ~20% and ~30% apoptosis in parental cells, respectively (Figure 3B). In contrast, niclosamide at the same concentration induced ~20%, ~30% and ~50% in chemo-resistant cells (Figure 3B). Time course analysis showed that niclosamide at 25 µM started to significantly induce apoptosis at 24 h and afterward (Figure 3C). Representative images of migration of osteosarcoma in the absence or presence of 5 µM shown in Figure 4A and C demonstrated a potent inhibitory effect (~90% inhibition) of niclosamide on osteosarcoma cell migration. In addition, niclosamide up to 1 µM caused ~60% and ~90% inhibition of migration in parental and chemo-resistant osteosarcoma cells, respectively (Figure 4B andD). Our data show that 1) niclosamide at sub-micromolar concentrations inhibited proliferation, induces apoptosis and suppresses migration in both parental and chemo-resistant cells; 2) chemo-resistant cells are more sensitive to niclosamide. 

### Niclosamide acts on osteosarcoma cells through suppressing β-catenin

**Figure 5 -:**
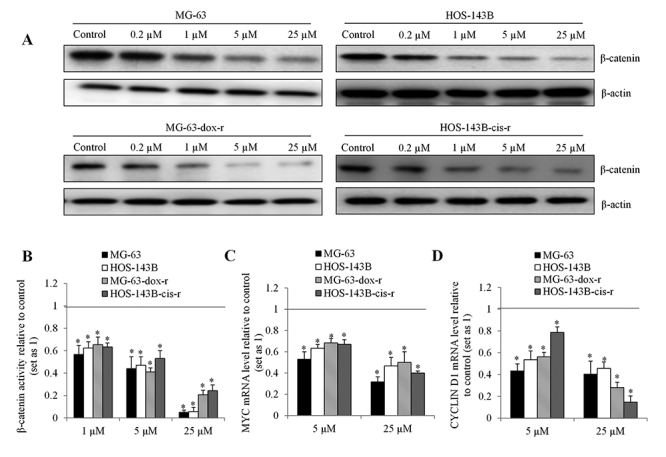
Niclosamide decreases **β-catenin** level and activity in parental and chemo-resistant osteosarcoma cells. (A) Western blot showing the decreased β-catenin level in MG-63, MG-63-dox-r, HOS-143B and HOS-143B-cis-r. Cells were treated with niclosamide for 24 h prior to western blot analyses. (B) Niclosamide significantly decreases β-catenin activity in parental and chemo-resistant osteosarcoma cells. TOP-flash luciferase reporter assay of osteosarcoma cells treated with niclosamide for 24 hours. (C and D) Niclosamide significantly decrease mRNA levels of MYC and CYCLIN D1. *, p<0.05, compared to control. Control was set up as 1. Unpaired Student’s t test was conducted.

Several studies indicated that niclosamide targets β-catenin to inhibit cancer cells (Arend et al., 2016; Jin et al., 2017; Wei et al., 2021; Guo et al., 2022). Given the importance of Wnt/β-catenin in osteosarcoma progression and chemoresistance (Ma et al., 2013; Hosseini et al., 2021), we investigated whether niclosamide acts on osteosarcoma via inhibiting Wnt/β-catenin. We measured β-catenin level and activity in osteosarcoma cells after niclosamide treatment. Immunoblotting analysis demonstrated that niclosamide decreased β-catenin protein level and the effective concentration started from 0.2 µM in all two parental and two chemo-resistant osteosarcoma cell lines (Figure 5A). Using TopFlash (TCL/LEF-Firefly luciferase) assay, we further showed that niclosamide decreased β-catenin activity (Figure 5B). Niclosamide at 25 µM gave near complete inhibition of β-catenin activity in MG-63 and HOS-143B cells. Consistent with the decreased β-catenin expression and activity, we observed the decreased mRNA levels of β-catenin-targeted genes: MYC and CYCLIN D1 (Figure 5C and D).

**Figure 6 -:**
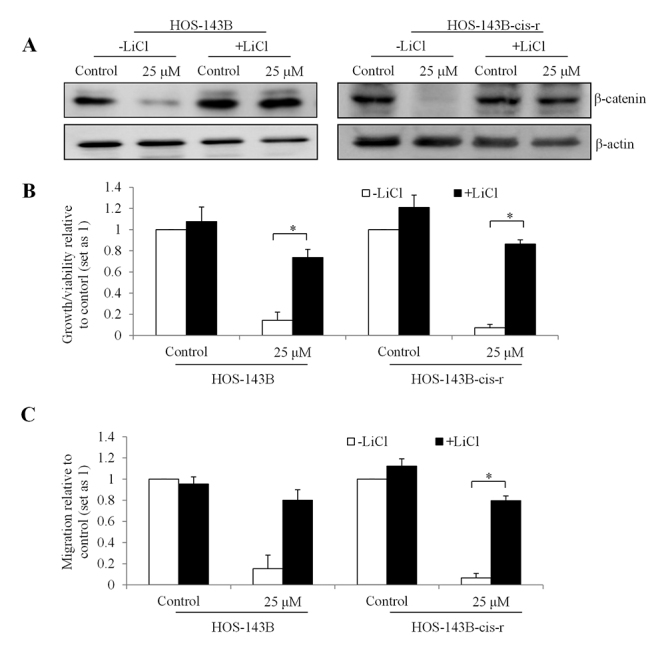
LiCl reverses the inhibitory effects of niclosamide in parental and chemo-resistant osteosarcoma cells. (A) Western blot showing no change on β-catenin level by niclosamide in HOS-143B and HOS-143B-cis-r cells treated with LiCl. The addition of LiCl reverses the inhibitory effects of niclosamide in growth/viability (B) and migration (C) in HOS-143B and HOS-143B-cis-r. *, p<0.05, compared to control. Control was set up as 1. One-way analysis of variance followed by Tukey’s HSD test was conducted for multiple comparisons.

To further confirm β-catenin as the molecular mechanism of action of niclosamide in osteosarcoma, we attempted to perform rescue experiments via reserving β-catenin levels using lithium chloride (LiCl) which is a Wnt activator by preventing β-catenin degradation (Galli et al., 2013). We did observe the decreased β-catenin in cells exposed to niclosamide but not to niclosamide and LiCl in HOS-143B and HOS-143B-cis-r cells (Figure 6A), suggesting that LiCl prevents niclosamide-induced β-catenin reduction. Of note, stabilization of β-catenin by LiCl significantly reversed the inhibitory effects of niclosamide on osteosarcoma cell growth/viability and migration (Figure 6B and C). These data confirm that niclosamide acts on parental and chemo-resistant osteosarcoma cells in a β-catenin-dependent manner.

### Chemo-resistant osteosarcoma cells display higher level of β-catenin activity compared to parental cells

**Figure 7 -:**
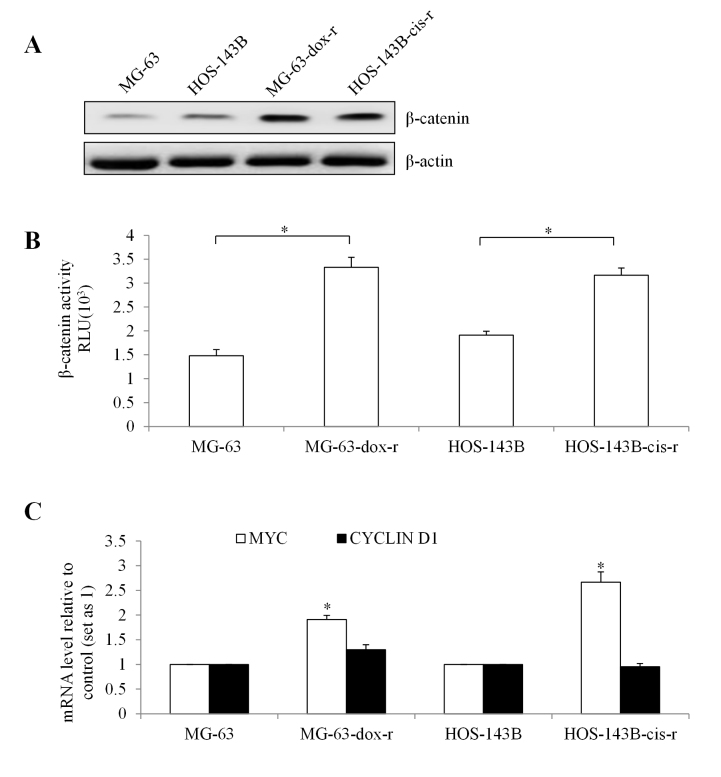
Chemo-resistant osteosarcoma cells display higher level of β-catenin signaling. (A) Western blot showing on β-catenin level in MG-63, MG-63-dox-r, HOS-143B and HOS-143B-cis-r cells. β-catenin activity (B) and mRNA levels of MYC and CYCLIN D1 (C) in MG-63, MG-63-dox-r, HOS-143B and HOS-143B-cis-r cells. *, p<0.05, compared to MG-63. MG-63 was set up as 1. One-way analysis of variance followed by Tukey’s HSD test and unpaired Student’s test were conducted.

To investigate the basis of chemo-resistant osteosarcoma cells hypersensitivity to niclosamide, we assessed baseline β-catenin expression level and activity in parental and chemo-resistant cells. MG-63-dox-r and HOS-143B-cis-r had higher levels of β-catenin compared to MG-63 and HOS-143B cells (Figure 7A). Consistent with the increased β-catenin expression, we observed the increased β-catenin activity in MG-63-dox-r and HOS-143B-cis-r (Figure 7B). We also observed the increased transcription level of MYC in chemo-resistant cells compared to parental cells (Figure 7C). However, there was no significant difference on CLYCIN D1 mRNA level between chemo-resistant and parental cells (Figure 7C). The significant increased levels of β-catenin and its activity in chemo-resistant osteosarcoma cells may provide a mechanism to explain hypersensitivity of chemo-resistant osteosarcoma cells to niclosamide. 

## Discussion

In this study, we evaluated niclosamide as a potential drug for overcoming chemo-resistance in patients with osteosarcoma. Niclosamide is an attractive candidate as it is already available for clinical use to treat parasitic infections in millions of people worldwide. It has been shown to be active in osteosarcoma cell lines, U2OS, HOS and MG63, and, furthermore, to prevent metastatic spread in the lungs in a mouse model of osteosarcoma (Li et al., 2015; Reddy et al., 2020; Yeh et al., 2022). The effect of niclosamide on osteosarcoma cells resistant to chemotherapy, a most relevant issue, had not yet been investigated. 

We first confirm and extend the previous findings by showing that niclosamide is effective in a panel of parental osteosarcoma cell lines. The cells lines HOS-143B-cis-r and MG63-dox-r were established for demonstrating the biological effects in a niclosamide model which is the most common mechanism of resistance reported in patients who became refractory to combinatory chemotherapy. Our findings show that niclosamide at sub-micromolar concentrations is active against cisplatin-resistant and doxorubicin-resistant osteosarcoma cells. This is supported by others’ work showing that niclosamide is able to overcome treatment resistance in prostate and breast cancer by enhancing the efficacy of abiraterone and cisplatin, respectively (Liu C et al., 2016; Liu J *et al.*, 2016). We previously also revealed that niclosamide sensitizes leukaemia cells to dasatinib (Liu Z *et al.*, 2016). The consistent findings from different cancers indicate that niclosamide is effective in targeting drug-resistant cancer cells. Similar to our previous finding that niclosamide targets leukemia cells but does not affect normal bone marrow cells (Liu Z *et al.*, 2016), the effective concentrations of niclosamide on osteosarcoma cells are minimally toxic to normal osteoblastic cells. The selectivity of niclosamide between malignant and normal osteoblastic cells is consistent with its safety profile in clinical trials (Burock et al., 2018) and demonstrates a therapeutic window of niclosamide for osteosarcoma. 

Unexpectedly, the anti-proliferative, anti-migratory and pro-apoptotic effects of niclosamide are stronger in chemo-resistant than parental osteosarcoma cells. The exact reason for this is unclear but our data suggests that chemo-resistant cells might be more dependent on β-catenin, which is the molecular target of niclosamide in osteosarcoma cells. Our mechanism studies show that niclosamide decreases β-catenin level and activity in parental and chemo-resistant cells, and furthermore, that β-catenin stabilization remarkably reverses the inhibitory effects of niclosamide. Of note, chemo-resistant cells have increased β-catenin level and activity, and increased expression of Wnt-targeted genes compared to parental cells. We thus speculate that chemoresistant cell lines might be more dependent on Wnt/β-catenin than sensitive lines. This speculation is supported by previous findings that β-catenin signalling is essential in chemo-resistant osteosarcoma, and targeting β-catenin is a therapeutic approach to sensitize osteosarcoma cells to chemotherapy (Ma et al., 2013; Hosseini et al., 2021). Although the activity of β-catenin and level of MYC were not lower in chemoresistant cell lines after niclosamide treatment than parental cell lines, the reduction of β-catenin activity and MYC level by niclosamide in chemoresistant cells is sufficient to inhibit growth and survival. It would be interesting to demonstrate that a cell line with extremely high endogenous level of β-catenin is resistant to chemotherapy but sensitive to niclosamide. We further demonstrated that MYC is the downstream target of niclosamide in osteosarcoma, most likely via the β-catenin/Myc axis. This is supported by previous findings that niclosamide dysregulates MYC in oral squamous cell carcinoma and lung cancer (Wang LH et al., 2018; Zuo et al., 2018). Given the important role of β-catenin in cancer stem cells, it is likely that niclosamide also targets osteosarcoma stem cells which serve as a reservoir for osteosarcoma relapse. Indeed, we previously showed that niclosamide can eliminate cancer stem cells (Liu Z et al., 2016).

In conclusion, our study confirms the anti-osteosarcoma activity of niclosamide. A significant finding of our work is that niclosamide is selective and effective in targeting chemo-resistant osteosarcoma cells. Our work also highlights the therapeutic value of β-catenin inhibition in overcoming osteosarcoma chemoresistance. Several clinical studies (NCT03123978; NCT02519582; NCT02687009; NCT03521232; NCT02807805) are ongoing to evaluate the efficacy of niclosamide against cancer. In lines with these efforts, our findings provide the preclinical evidence to initialize clinical trials to investigate the efficacy of niclosamide in osteosarcoma patients who have become refractory to chemotherapy. 
